# Global prevalence of congenital anencephaly: a comprehensive systematic review and meta-analysis

**DOI:** 10.1186/s12978-022-01509-4

**Published:** 2022-10-17

**Authors:** Nader Salari, Behnaz Fatahi, Reza Fatahian, Payam Mohammadi, Adibeh Rahmani, Niloofar Darvishi, Mona Keivan, Shamarina Shohaimi, Masoud Mohammadi

**Affiliations:** 1 Department of Biostatistics, School of Health, Kermanshah University of Medical Sciences, Kermanshah, Iran; 2 Student Research Committee, Kermanshah University of Medical Sciences, Kermanshah, Iran; 3 Department of Neurosurgery, School of Medicine, Kermanshah University of Medical Sciences, Kermanshah, Iran; 4 Department of Neurology, School of Medicine, Kermanshah University of Medical Sciences, Kermanshah, Iran; 5 Julius Wolff Institute, Chatite, Berlin, Germany; 6 Student Research Committee, School of Medicine, Kermanshah University of Medical Sciences, Kermanshah, Iran; 7 Department of Biology, Faculty of Science, University Putra Malaysia, Serdang, Selangor Malaysia; 8 Cellular and Molecular Research Center, Gerash University of Medical Sciences, Gerash, Iran

**Keywords:** Prevalence, Neural tube defects, Anencephaly, Systematic review and meta-analysis

## Abstract

**Background:**

Anencephaly is a fatal congenital anomaly characterized by the absence of brain hemispheres and cranial arch. Timely preventive measures can be taken by knowing the exact prevalence of this common neural tube defect; thus, carried out through systematic review and meta-analysis, the present study was conducted to determine the worldwide prevalence, incidence and mortality of anencephaly.

**Methods:**

Cochran’s seven-step instructions were used as the guideline. Having determined the research question and inclusion and exclusion criteria, we studied MagIran, SID, Science Direct, WoS, Web of Science, Medline (PubMed), Scopus, and Google Scholar databases. Moreover, the search strategy in each database included using all possible keyword combinations with the help of “AND” and “OR” operators with no time limit to 2021. The I^2^ test was used to calculate study heterogeneity, and Begg and Mazumdar rank correlation tests were employed to assess the publication bias. Data were analyzed by Comprehensive Meta-Analysis software (Version 2).

**Results:**

In this study, the statements of Preferred Reporting Items for Systematic Reviews and Meta-Analyzes (PRISMA) were used. In the first stage, 1141 articles were found, of which 330 duplicate studies were omitted. 371 articles were deleted based on the inclusion and exclusion criteria by reviewing the title and abstract of the study. 58 articles were removed by reviewing the full text of the article because it was not relevant to the research. 360 studies with a sample size of 207,639,132 people were considered for the meta-analysis. Overall estimate of the prevalence, incidence and attenuation of anencephaly worldwide were 5.1 per ten thousand births (95% confidence interval 4.7–5.5 per ten thousand births), 8.3 per ten thousand births (95% confidence interval 5.5–9.9 per ten thousand births), 5.5 per ten thousand births (95% confidence interval 1.8–15 per ten thousand births) respectively the highest of which according to the subgroup analysis, belonged to the Australian continent with 8.6 per ten thousand births (95% confidence interval 7.7–9.5 per ten thousand births).

**Conclusion:**

The overall prevalence of anencephaly in the world is significant, indicating the urgent need for preventive and treating measures.

## Plain Language summary

Anencephaly is a fatal congenital anomaly characterized by the absence of brain hemispheres and cranial arch. Cochran’s seven-step instructions were used as the guideline. Having determined the research question and inclusion and exclusion criteria, we studied MagIran, SID, Science Direct, WoS, Web of Science, Medline (PubMed), Scopus, and Google Scholar databases. Moreover, the search strategy in each database included using all possible keyword combinations with the help of “AND” and “OR” operators with no time limit to 2021. Out of 1141 initial articles found, and after excluding repetitive ones in various databases and those irrelevant to inclusion criteria, 360 studies with a sample size of 207,639,132 people were considered for the meta-analysis. Overall estimate of the prevalence, incidence and attenuation of anencephaly worldwide were 5.1 per ten thousand births (95% confidence interval 4.7–5.5 per ten thousand births), 8.3 per ten thousand births (95% confidence interval 5.5–9.9 per ten thousand births), 5.5 per ten thousand births (95% confidence interval 1.8–15 per ten thousand births) respectively the highest of which according to the subgroup analysis, belonged to the Australian continent with 8.6 per ten thousand births (95% confidence interval 7.7–9.5 per ten thousand births). The overall prevalence of anencephaly in the world is significant, indicating the urgent need for preventive and treating measures.

## Background

Neural Tube Defects (NTDs) are considered the most common congenital anomalies of the central nervous system (CNS) [1], and the second most serious ones after inborn heart defects [2]. Non-spontaneous neural tube closure between the 3rd and 4th weeks of intrauterine growth is considered as the leading cause of this defect [1]. Regarding the etiology of these defects, most cases are attributed to the interaction between different genes and environmental factors, known as a multifactorial inheritance [3]. Studies indicate that immediate family members are more at risk compared to others; in other words, if a child is born with NTD, the risk of recurrence in future pregnancies is between 25 and 50 times higher than in general cases [4, 5, 6]. Moreover, diabetes mellitus, using valproic acid to treat epilepsy during pregnancy, obesity, zinc deficiency, hyperthermia, and folate deficiency are all predisposing factors for neural tube defects [7, 8].

Though being significantly various in different geographical areas, the incidence of NTD is generally around 1 in 1000 live births or (NTD affects about 1 in 1000 live births on average, however this varies greatly by area.) [4, 9]. Pathologically, neural tube defects vary from a small, uncomplicated opening in the posterior canal of the vertebrae to the failure of the entire neural tube to close, leading to the most severe type of defect that is craniorachischisis [10]. The most recurring cases include anencephaly, spina bifida, and encephalocele [10].

Anencephaly is a fatal congenital malformation characterized by the absence of hemispheres of the brain and cranial arch [11]. Anencephaly is the most common CNS disorder in the Western world, occurring 37 times more frequently in women than men [12]. Babies born with such defects generally die at birth or shortly thereafter while newborns with spina bifida and encephalocele require special medical care and surgery to survive [13]. Prevalence of anencephaly mortality (100%), compared to Spina bifida (7%) and encephalocele (46%), is significantly higher [14]; thus, anencephaly is considered as a taxing burden on public health worldwide that may lead to significant human resources loss [15].

Frog-like appearance, short neck, bulging eyes, and large tongue are characteristic features of infants with anencephaly [16]. About 12% of cases of anencephaly are associated with other structural abnormalities [17], including Cleft lip, cleft palate, clubfoot and omphalocele (Anencephaly is linked to additional structural abnormalities in around 12% of cases [17], such as cleft lip, cleft palate, clubfoot, and omphalocele) [16]. Anencephaly was the first congenital anomaly to be detected by ultrasound, and it can be diagnosed at weeks 12–13 of pregnancy while preventive measures include controlling known risk factors and offering medical counseling to couples about termination of pregnancy [16]. Previous studies have demonstrated that anencephaly is a multifactorial process that is controlled by genes and numerous other environmental factors. However, recent studies reveal that folic acid supply before and in the early stages of pregnancy (1 to 3 months before pregnancy and up to 12 weeks of gestation) can dramatically prevent anencephaly and reduce its prevalence by 50–70% [18]. The U.S. Public Health Service and the Food and Nutrition Council of the Institute of Medicine, along with the National Research Council, recommend that all women of childbearing potential can take 0.4 mg of folic acid daily to reduce the risk of developing neural tube defects [19, 20].

Annually, about 300,000 babies are born with neural tube defects, resulting in 88,000 deaths and 8.6 million lifelong disabilities [21]. The occurrence of anencephaly varies over time and geographically. For instance, the prevalence of this defect in northern Iran in 1998–2005 was estimated at 12 per 10,000 births [22] while In Texas, the United States, 2.81 per 10,000 births during 1999–2003 were reported [23]. The prevalence of anencephaly based on data collected from (EUROCAT) member countries during the years 2000 and 2010, was estimated at 3.52 per 10,000 births [24].

Considering the importance of anencephaly as the most severe type of neural tube defect, and its detrimental effects on the quantity and quality of patients’ and parents’ life, and regarding the serious health, psychological, social and economic costs for the individual and society, accurate identification of patients is of great importance to organize health care services and implement preventive measures. In addition, because of various statistics on the prevalence of anencephaly and the worldwide absence of a comprehensive investigation capable of analyzing the outcomes of these studies, the present research was conducted through a systematic review and meta-analysis to shed light on the prevalence, incidence and mortality of anencephaly worldwide.

## Methods

The present systematic review and meta-analysis was conducted based on the Cochrane 7-step approach, including: research question selection, inclusion and exclusion criteria, article identification, study selection, study quality evaluation, data extraction, and analysis and interpretation of findings [25]. In this study, the statements of Preferred Reporting Items for Systematic Reviews and Meta-Analyzes (PRISMA) were used [26].

### Research question and keyword determination

According to the research question “How has the prevalence, incidence and mortality of anencephaly changed worldwide?” the following were defined:

The study population (Population) included patients with anencephaly, result (Outcome) comprised the prevalence of anencephaly, date of publishing the first related article until March 23, 2021 was specified as the time range (Time or Duration), and type of study (study design) included cross-sectional studies (descriptive, descriptive-analytical). Keywords were extracted from the MeSH browser. Keywords related to the studied population (P): Anencephaly, Congenital Absence of Brain, Anencephalus, Anencephalia, Incomplete Anencephaly, Partial Anencephaly, Hemicranial Anencephaly, Aprosencephaly and Keywords related to outcome were (O), Prevalence, outbreak.

### Inclusion and exclusion criteria according to the research question

Cross-sectional population-based studies (descriptive, descriptive-analytical) that reported the prevalence of anencephaly in different parts of the world, published in Persian and English with full texts available included in the study. Analytical, interventional, conferential, and group-case studies irrelevant to the research question and studies that were not in English or did not have English abstracts were excluded from the investigation.

### Article identification

To review the literature, two Persian databases, including MagIran and SID, and four international ones, Science Direct, Web of Science (WoS), Medline (PubMed), and Scopus, were selected. The Google Scholar scientific search engine was considered for final review while no time limit was set for the search to retrieve relevant results; thus, all articles published up to March 23, 2021 were reviewed. Searching was limited to studies published in Persian and English and strategy in each database was determined using Advanced Search (Advance Search) with the help of all possible keyword combinations with the help of AND and OR operators. For example, searching strategy in the PubMed database was determined as follows:

(prevalence [Title/Abstract] OR outbreak [Title/Abstract]) AND (Anencephaly [Title/Abstract] OR Congenital Absence of Brain [Title/Abstract] OR Anencephalus [Title/Abstract] OR Anencephalia [Title/Abstract] OR Incomplete Anencephaly [Title/Abstract] OR Partial Anencephaly [Title/Abstract] OR Hemicranial Anencephaly [Title/Abstract] OR Aprosencephaly [Title/Abstract]).

In order to access the latest published studies, an alert was created on a number of important databases, including PubMed and Scopus, to check if new articles were published during the study. Also, in order to access all related studies, the sources of articles that met the inclusion criteria were manually reviewed. To avoid error, all steps of article search, study selection, qualitative evaluation and data extraction were performed independently by two researchers (BF and ND). If there was a difference of opinion among the researchers regarding the inclusion of the article in the study, in order to avoid the risk of biased selections for specific studies, first a final agreement was reached through discussion and in some cases with the participation and opinion of a third party (MM).

### Selection of studies based on inclusion and exclusion criteria

The information of all articles found in each database was transferred to EndNote X8 software. After completing the search in all the databases, duplicate articles were excluded. Then, in order to avoid the risk of prejudice in selecting studies, the names of the authors and the titles of the journals of the articles were removed and a checklist was prepared based on the titles and abstracts of the studies. In the next step, two authors (N.D. and B.F.) independently examined the title and abstract of the research and eliminated irrelevant papers based on the inclusion and exclusion criteria. Studies with no full text were not considered for the systematic review and meta-analysis process. The full text of all remaining articles was then evaluated. Studies that did not meet the inclusion criteria based on the research question were out listed.

### Qualitative evaluation of studies

Qualitative evaluation of studies was performed using the Newcastle–Ottawa Scale, the NOS assigns a maximum of 9 points for the three areas of study group selection, group comparison, and exposure and outcome for the case and group studies [27]. Based on this, articles were classified as high quality (≥ 5) and low quality (< 5).

### Extracting the data

After selecting the studies to enter the systematic review and meta-analysis process, the data were extracted and the studies were summarized. An electronic checklist was prepared for this purpose. The various items on the checklist included: the surname of the first author, year of publication and year of the report, sample size, number of patients, prevalence, incidence and mortality of patients.

### Statistical analysis

To analyze and combine the results of different studies, in each study, the prevalence of anencephaly was considered as the probability of two-sentence distribution and its variance was calculated through two-sentence distribution. Heterogeneity of studies was assessed using I^2^ test. A Random effect model was used in case of I^2^ index above 50%. In this model, parameter changes between studies are also considered in the calculations, so it can be said that the results of this model in heterogeneous conditions can be more generalized than the model with a fixed effect. Due to the large sample size investigated in the study, Begg and Mazumdar rank correlation test was used at a significance level of 0.1 to check the publication bias. Data were analyzed using Comprehensive Meta-Analysis (Version 2) software.

## Results

### Summary of how articles entered the meta-analysis

**Fig. 1:**
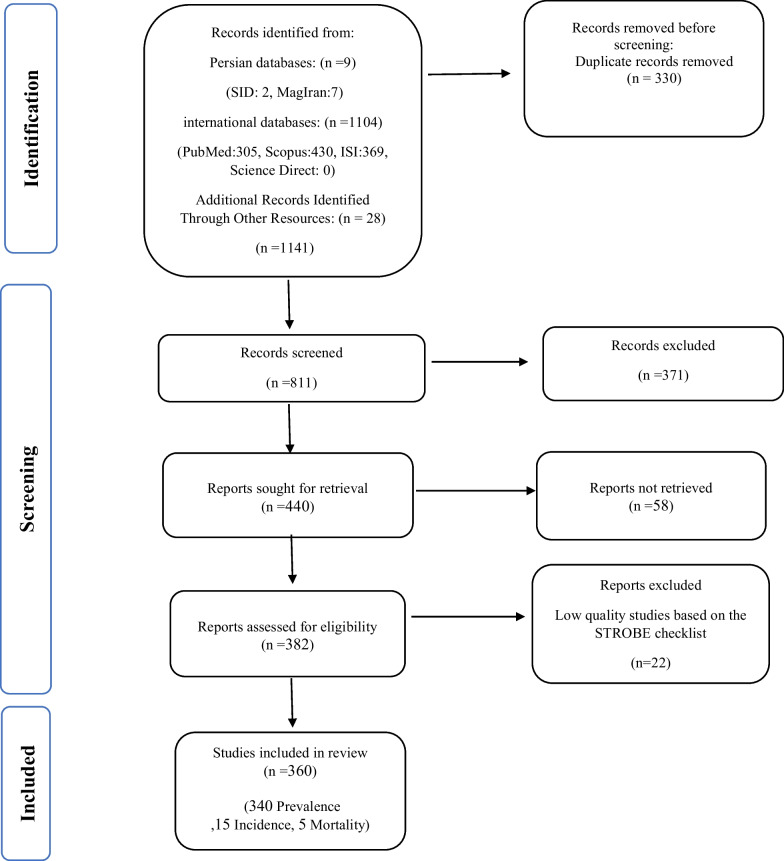
Preferred reporting items for systematic reviews and meta-analyses (PRISMA 2020) flow diagram

In this study, the statements of Preferred Reporting Items for Systematic Reviews and Meta-Analyzes (PRISMA) were used [26]. In the first stage, 1141 articles (1104 articles in international, 9 articles in Persian databases and 28 studies in reviewing article sources) were found, of which 330 duplicate studies were omitted. 811 studies entered the screening stage and 371 articles were deleted based on the inclusion and exclusion criteria by reviewing the title and abstract of the study. In the next stage (competency assessment), out of the remaining 440 studies from the screening stage, 58 articles were removed by reviewing the full text of the article because it was not relevant to the research. The quality evaluation of 382 articles included in this study was performed using the STROBE checklist, of which 22 studies had poor methodological quality and were deleted. Thus, 360 related studies entered the process of systematic review and meta-analysis (Fig. 1) [28].

### General characteristics of the studies:

The total sample size of the prevalence studies was 169,407,738 people. The studies were published between 1969 and March 23, 2021. The lowest sample size was related to the study of Castilla-17 et al. (1985) with 1623 people in [29] Colombia and the highest sample size was related to the study of James et al. (1993) with 15,487,449 people in the USA [30]. The surname of the first author, year of publication and year of reporting, place of study, maternal age, sample size, number of cases, prevalence, incidence and attenuation of anencephaly reported in Tables 1, 2 and 3.

**Table 1:** Summary of study specifications (prevalence of anencephaly)

First author, year, References	Report year	Continent	Country	Sample size	Number of patients with Anencephaly
Gong-1, 2017, [1]	2006	Asia	China	306,734	227
Gong-2, 2017, [1]	2007	Asia	China	341,432	244
Gong-3, 2017, [1]	2008	Asia	China	330,414	186
Gong-4, 2017, [1]	2009	Asia	China	321,353	166
Gong-5, 2017, [1]	2010	Asia	China	307,826	168
Gong-6, 2017, [1]	2011	Asia	China	304,079	158
Gong-7, 2017, [1]	2012	Asia	China	353,108	145
Gong-8, 2017, [1]	2013	Asia	China	321,171	141
Gong-9, 2017, [1]	2014	Asia	China	364,400	108
Gong-10, 2017, [1]	2015	Asia	China	298,437	55
PEI, 2009, [2]	2004–2006	Asia	China	4175	28
Afshar, 2006, [3]	1997–2001	Asia	Iran	16,785	23
Golalipour-1, 2007, [4]	1998–2003	Asia	Iran	37,951	43
Li, 2006, [5]	2003	Asia	China	11,534	76
LIAN, 1987, [6]	1970–1984	Asia	China	208,801	461
Golalipour-2, 2010, [7]	1998–2005	Asia	Iran	30,639	35
Xie, 2020, [8]	2015–2018	Asia	China	705,395	188
Khattak, 2010, [9]	2007	Asia	SWAT	5560	63
Golalipour-3, 2010, [10]	1998–2005	Asia	Iran	49,534	56
Zhang-1, 2012, [11]	2005–2008	Asia	China	62,443	43
Jung-1, 1999, [12]	1993	Asia	Korea	601,376	156
Jung-2, 1999, [12]	1994	Asia	Korea	601,459	255
Jaruratanasirikul, 2014, [13]	2009–2012	Asia	Thailand	148,759	12
Zhu-1, 2012, [14]	2006	Asia	China	643,987	407
Zhu-2, 2012, [14]	2007	Asia	China	777,397	454
Zhu-3, 2012, [14]	2008	Asia	China	843,920	465
Jin-1, 2017, [15]	2006	Asia	China	22,559	16
Jin-2, 2017, [15]	2007	Asia	China	26,874	13
Jin-3, 2017, [15]	2008	Asia	China	28,291	19
Jin-4, 2017, [15]	2009	Asia	China	27,916	20
Jin-5, 2017, [15]	2010	Asia	China	26,973	12
Jin-6, 2017, [15]	2011	Asia	China	28,424	9
Jin-7, 2017, [15]	2012	Asia	China	32,489	13
Kant, 2017, [16]	2001–2014	Asia	India	26,946	33
Liu, 2007, [17]	1996–2004	Asia	China	99,888	42
Ebrahimi, 2013, [18]	2005–2011	Asia	Iran	14,034	59
Ghavami, 2011, [19]	2005–2008	Asia	Iran	22,500	18
Kondo-1, 2019, [20]	2014	Asia	Japan	156,791	13
Kondo-2, 2019, [20]	2015	Asia	Japan	158,347	13
Tiwari, 2020, [21]	2014	Asia	India	14,681	19
IMAIZUMI-1, 1991, [22]	1948–1958	Asia	Japan	27,891	27
IMAIZUMI-2, 1991, [22]	1959–1969	Asia	Japan	40,715	22
IMAIZUMI-3, 1991, [22]	1970–1980	Asia	Japan	39,506	28
IMAIZUMI-4, 1991, [22]	1981–1990	Asia	Japan	23,884	17
Zhang-2, 2017, [23]	2006–2015	Asia	China	3,248,954	1600
Seto-1, 2003, [24]	1981–1990	Asia	Japan	136,846	39
Seto-2, 2003, [24]	1991–2000	Asia	Japan	117,332	7
Fakheri, 2004, [25]	1996–2001	Asia	Iran	81,538	106
PourIsa, 2005, [26]	1997–2003	Asia	Iran	21,074	29
Golalipour-4, 2004, [27]	1997–2001	Asia	Iran	26,280	39
Stoll-1, 2006, [28]	1988–1992	Europe	France	68,326	9
Stoll-2, 2006, [28]	1993–1995	Europe	France	39,286	4
Stoll-3, 2006, [28]	1996–2002	Europe	France	95,058	10
RICHARDS, 1972, [31]	1964–1966	Europe	Wales	92,980	2145
Stoll-4, 2011, [32]	1979–2008	Europe	France	402,532	182
Szabó-1, 2013, [33]	1980–1991	Europe	Hungary	209,762	64
Szabó-2, 2013, [33]	1994–2005	Europe	Hungary	155,978	29
Pietrzyk-1, 1983, [34]	1970–1972	Europe	Poland	33,766	9
Pietrzyk-2, 1983, [34]	1979- 1981	Europe	Poland	46,818	11
McDonnell-1, 1999, [35]	1980–1994	Europe	East Ireland	320,750	322
Boyd-1, 2000, [36]	2000	Europe	Denmark	8788	2
Boyd-2, 2000, [36]	2000	Europe	Netherlands	81,980	18
Boyd-3, 2000, [36]	2000	Europe	Austria	29,026	3
Boyd-4, 2000, [36]	2000	Europe	Croatia	10,718	2
Boyd-5, 2000, [36]	2000	Europe	France	60,705	15
Boyd-6, 2000, [36]	2000	Europe	Germany	18,280	7
Boyd-7, 2000, [36]	2000	Europe	Italy	204,178	34
Boyd-8, 2000, [36]	2000	Europe	Lithuania	95,469	29
Boyd-9, 2000, [36]	2000	Europe	Spain	38,166	14
Boyd-10, 2000, [36]	2000	Europe	Ukraine	44,761	11
Boyd-11, 2000, [36]	2000	Europe	UK	78,695	31
Salvador, 2011, [37]	1992–2006	Europe	Spain	197,003	87
DOLK-1, 1991, [38]	19,980–1987	Europe	UK& Ireland	577,989	739
DOLK-2, 1991, [38]	19,980–1986	Europe	Europe & Malta	378,849	184
Khoshnood-1, 2015, [39]	1991–2009	Europe	Austria	216,196	40
Khoshnood-2, 2015, [39]	1991–2011	Europe	Belgium	601,565	182
Khoshnood-3, 2015, [39]	2000–2009	Europe	Czech Republic	1,029,247	245
Khoshnood-4, 2015, [39]	1991–2010	Europe	Croatia	131 525	18
Khoshnood-5, 2015, [39]	1991–2011	Europe	Denmark	115 846	44
Khoshnood-6, 2015, [39]	1993–2010	Europe	Finland	1,070,940	314
Khoshnood-7, 2015, [39]	1991–2011	Europe	France	666,353	347
Khoshnood-8, 2015, [39]	1991–2011	Europe	Germany	360,801	95
Khoshnood-9, 2015, [39]	1998–2010	Europe	Hungary	1,260,719	256
Khoshnood-10, 2015, [39]	1991–2011	Europe	Ireland	702,747	244
Khoshnood-11, 2015, [39]	1991–2011	Europe	Italy	1,215,306	217
Khoshnood-12, 2015, [39]	1991–2010	Europe	Malta	88,573	25
Khoshnood-13, 2015, [39]	1991–2011	Europe	Netherlands	401,404	108
Khoshnood-14, 2015, [39]	1999–2011	Europe	Norway	775,060	282
Khoshnood-15, 2015, [39]	1999–2010	Europe	Poland	440,163	71
Khoshnood-16, 2015, [39]	1991–2010	Europe	Portugal	316,853	62
Khoshnood-17, 2015, [39]	1991–2010	Europe	Spain	361,416	189
Khoshnood-18, 2015, [39]	1991–2011	Europe	Switzerland	159,273	62
Khoshnood-19, 2015, [39]	1991–2011	Europe	UK	2,556,075	1361
Loane, 2009, [40]	2000–2004	Europe	UK	1,740,718	40
Peake, 2021, [41]	2006–2011	Europe	UK	1,351,405	673
Boyd-12, 2011, [42]	2005–2006	Europe	UK	601,545	366
Poretti, 2008, [43]	2001–2007	Europe	Switzerland	10,769,230	22
Obeid-1, 2015, [44]	2000–2010	Europe	Europe	9,161,189	3221
Obeid-2, 2015, [44]	2000–2010	Europe	Germany	227,781	56
GARNE, 2005, [45]	1995–1999	Europe	17 European regions	1,198,519	498
CADAS, 1978, [46]	1955–1965	Europe	Greece	74,390	49
Loncarek-1, 2001, [47]	1963	Europe	Croatia	2946	1
Loncarek-2, 2001, [47]	1966	Europe	Croatia	2988	1
Loncarek-3, 2001, [47]	1967	Europe	Croatia	2974	2
Loncarek-4, 2001, [47]	1971	Europe	Croatia	3582	1
Loncarek-5, 2001, [47]	1972	Europe	Croatia	3522	1
Loncarek-6, 2001, [47]	1973	Europe	Croatia	3580	1
Loncarek-7, 2001, [47]	1974	Europe	Croatia	3612	1
Loncarek-8, 2001, [47]	1975	Europe	Croatia	3692	1
Loncarek-9, 2001, [47]	1979	Europe	Croatia	4174	1
Loncarek-10, 2001, [47]	1980	Europe	Croatia	4242	1
Loncarek-11, 2001, [47]	1983	Europe	Croatia	4042	3
Loncarek-12, 2001, [47]	1988	Europe	Croatia	3655	1
Loncarek-13, 2001, [47]	1989	Europe	Croatia	3504	2
Loncarek-14, 2001, [47]	1992	Europe	Croatia	3647	1
Loncarek-15, 2001, [47]	1993	Europe	Croatia	3468	1
Loncarek-16, 2001, [47]	1994	Europe	Croatia	3326	1
Loncarek-17,2001, [47]	1996	Europe	Croatia	3412	1
Loncarek-18, 2001, [47]	1998	Europe	Croatia	3017	1
EUROCAT GROUP-1, 1991, [48]	1980–1986	Europe	Republic of Ireland	183,278	242
EUROCAT GROUP-2, 1991, [48]	1980–1986	Europe	UK	467,437	597
EUROCAT GROUP-3, 1991, [48]	1980–1986	Europe	Belgium	57,352	31
EUROCAT GROUP-4, 1991, [48]	1980–1986	Europe	Netherlands	50,437	33
EUROCAT GROUP-5, 1991, [48]	1980–1986	Europe	Denmark	32,648	17
EUROCAT GROUP-6, 1991, [48]	1980–1986	Europe	France	349,737	143
EUROCAT GROUP-7, 1991, [48]	1980–1986	Europe	Italy	63,261	28
EUROCAT GROUP-8, 1991, [48]	1980–1986	Europe	Malta	22,225	13
EUROCAT GROUP-9, 1987, [49]	1980–1983	Europe	Republic of Ireland	109,276	168
EUROCAT GROUP-10, 1987, [49]	1980–1983	Europe	UK	244,955	309
EUROCAT GROUP-11, 1987, [49]	1980–1983	Europe	Denmark	18,533	8
EUROCAT GROUP-12, 1987, [49]	1980–1983	Europe	Netherlands	23,150	13
EUROCAT GROUP-13, 1987, [49]	1980–1983	Europe	Belgium	60,034	25
EUROCAT GROUP-14, 1987, [49]	1980–1983	Europe	France	143,878	69
EUROCAT GROUP-15, 1987, [49]	1980–1983	Europe	Luxembourg	9148	3
EUROCAT GROUP-16, 1987, [49]	1980–1983	Europe	Germany	21,985	9
EUROCAT GROUP-17, 1987, [49]	1980–1983	Europe	Italy	135,662	28
Smithells, 1989, [50]	1985–1986	Europe	UK	97,101	67
Corona-Rivera-1, 2021, [51]	1991–2002	Europe	Mexico	95,454	21
Corona-Rivera-2, 2021, [51]	2003–2019	Europe	Mexico	171,795	67
Stone-1, 1988, [52]	1974	Europe	Scotland	14,880	33
Stone-2, 1988, [52]	1975	Europe	Scotland	14,398	39
Stone-3, 1988, [52]	1976	Europe	Scotland	12,889	34
Stone-4, 1988, [52]	1977	Europe	Scotland	12,487	28
Stone-5, 1988, [52]	1978	Europe	Scotland	12,491	30
Stone-6, 1988, [52]	1979	Europe	Scotland	13,339	29
Stone-7, 1988, [52]	1980	Europe	Scotland	13,438	24
Stone-8, 1988, [52]	1981	Europe	Scotland	13,491	19
Stone-9, 1988, [52]	1982	Europe	Scotland	12,884	19
Stone-10, 1988, [52]	1983	Europe	Scotland	12,661	19
Stone-11, 1988, [52]	1984	Europe	Scotland	12,783	14
Stone-12, 1988, [52]	1985	Europe	Scotland	13,089	15
CARSTAIRS-1, 1984, [53]	1971	Europe	Scotland	87,883	224
CARSTAIRS-2, 1984, [53]	1972	Europe	Scotland	79,603	185
CARSTAIRS-3, 1984, [53]	1973	Europe	Scotland	75,265	181
CARSTAIRS-4, 1984, [53]	1974	Europe	Scotland	70,943	156
CARSTAIRS-5, 1984, [53]	1975	Europe	Scotland	68,708	140
CARSTAIRS-6, 1984, [53]	1976	Europe	Scotland	65,524	89
CARSTAIRS-7, 1984, [53]	1977	Europe	Scotland	62,895	66
CARSTAIRS-8, 1984, [53]	1978	Europe	Scotland	64,819	57
CARSTAIRS-9, 1984, [53]	1979	Europe	Scotland	68,841	47
CARSTAIRS-10, 1984, [53]	1980	Europe	Scotland	69,355	32
CARSTAIRS-11, 1984, [53]	1981	Europe	Scotland	69,490	19
CARSTAIRS-12, 1984, [53]	1982	Europe	Scotland	66,582	13
Rankin-1, 2000, [54]	1984	Europe	UK	39,357	27
Rankin-2, 2000, [54]	1985	Europe	UK	41,175	33
Rankin-3, 2000, [54]	1986	Europe	UK	40,541	27
Rankin-4, 2000, [54]	1987	Europe	UK	40,700	35
Rankin-5, 2000, [54]	1988	Europe	UK	40,428	33
Rankin-6, 2000, [54]	1989	Europe	UK	39,411	36
Rankin-7, 2000, [54]	1990	Europe	UK	40,966	30
Rankin-8, 2000, [54]	1991	Europe	UK	41,484	26
Rankin-9, 2000, [54]	1992	Europe	UK	40,316	41
Rankin-10, 2000, [54]	1993	Europe	UK	38,960	26
Rankin-11, 2000, [54]	1994	Europe	UK	35,380	21
Rankin-12, 2000, [54]	1995	Europe	UK	34,487	32
Rankin-13, 2000, [54]	1996	Europe	UK	34,024	21
Fleurke-Rozema, 2015, [55]	2008–2013	Europe	Netherlands	203,703	110
Sever-1, 1982, [56]	1966	America	USA	124,467	66
Sever-2, 1982, [56]	1967	America	USA	124,441	55
Sever-3, 1982, [56]	1968	America	USA	126,637	61
Sever-4, 1982, [56]	1969	America	USA	131,343	82
Sever-5, 1982, [56]	1970	America	USA	134,045	65
Sever-6, 1982, [56]	1971	America	USA	117,324	59
Sever-7, 1982, [56]	1972	America	USA	107,094	60
Limb-1, 1994, [57]	1972–1974	America	USA	18,155	17
Limb-2, 1994, [57]	1979–1981	America	USA	21,436	10
Limb-3, 1994, [57]	1982–1984	America	USA	25,218	11
Limb-4, 1994, [57]	1985–1987	America	USA	30,217	16
Limb-5, 1994, [57]	1988–1990	America	USA	31,290	20
Groisman-1, 2019, [58]	2016	America	Argentina	305,452	57
Rowland, 2006, [59]	1968–2002	America	USA	1,164,865	431
Krajewski, 2021, [60]	2007–2010	America	USA	1,610,709	433
Bronberg, 2020, [61]	2010–2016	America	Argentina	228,208	111
Carmichael, 2004, [62]	1989–1997	America	USA	2,234,846	535
Shaw, 2002, [63]	1985–1997	America	USA	1,303,306	197
Estevez-Ordonez, 2017, [64]	2010–2015	America	Honduras	123,903	30
Biggar-1, 1976, [65]	1918	America	USA	7199	3
Biggar-2, 1976, [65]	1919	America	USA	6973	1
Biggar-3, 1976, [65]	1920	America	USA	7153	10
Biggar-4, 1976, [65]	1921	America	USA	7272	4
Biggar-5, 1976, [65]	1922	America	USA	6905	3
Biggar-6, 1976, [65]	1923	America	USA	7256	7
Biggar-7, 1976, [65]	1924	America	USA	5967	3
Biggar-8, 1976, [65]	1925	America	USA	6925	2
Biggar-9, 1976, [65]	1926	America	USA	6393	3
Biggar-10, 1976, [65]	1927	America	USA	6717	8
Biggar-11, 1976, [65]	1928	America	USA	6370	5
Biggar-12, 1976, [65]	1929	America	USA	6116	7
Biggar-13, 1976, [65]	1930	America	USA	5872	2
Biggar-14, 1976, [65]	1931	America	USA	5632	8
Biggar-15, 1976, [65]	1932	America	USA	5574	6
Biggar-16, 1976, [65]	1933	America	USA	5065	7
Biggar-17, 1976, [65]	1934	America	USA	5127	10
Biggar-18, 1976, [65]	1935	America	USA	5101	6
Biggar-19, 1976, [65]	1936	America	USA	5056	8
Biggar-20, 1976, [65]	1937	America	USA	5314	8
Biggar-21, 1976, [65]	1938	America	USA	5613	7
Sargiotto, 2015, [66]	2009–2013	America	Argentina	703,325	212
Pacheco, 2009, [67]	2000–2006	America	Brasil	161,341	34
Janerich-1, 1973, [68]	1945–1947	America	USA	407,326	463
Janerich-2, 1973, [68]	1948–1950	America	USA	454,206	476
Janerich-3, 1973, [68]	1951–1953	America	USA	510,601	397
Janerich-4, 1973, [68]	1954–1956	America	USA	565.391	398
Janerich-5, 1973, [68]	1957–1959	America	USA	601,196	375
Janerich-6, 1973, [68]	1960–1962	America	USA	605,336	392
Janerich-7, 1973, [68]	1963–1965	America	USA	574,662	376
Janerich-8, 1973, [68]	1966–1968	America	USA	506,706	337
Janerich-9, 1973, [68]	1969–1971	America	USA	499,131	248
Jorde, 1984, [69]	1940–1979	America	USA	979,873	374
Castilla-1, 1985, [29]	1967	America	South America	12,430	7
Castilla-2, 1985, [29]	1968	America	South American	33,874	8
Castilla-3, 1985, [29]	1969	America	South American	42,874	7
Castilla-4, 1985, [29]	1970	America	South American	51,535	11
Castilla-5, 1985, [29]	1971	America	South American	47,156	9
Castilla-6, 1985, [29]	1972	America	South American	50,786	13
Castilla-7, 1985, [29]	1973	America	South American	65,009	13
Castilla-8, 1985, [29]	1974	America	South American	84,961	31
Castilla-9, 1985, [29]	1975	America	South American	65,214	11
Castilla-10, 1985, [29]	1976	America	South American	77,992	22
Castilla-11, 1985, [29]	1977	America	South American	67,432	19
Castilla-12, 1985, [29]	1978	America	South American	72,231	21
Castilla-13, 1985, [29]	1979	America	South American	68,645	20
Castilla-14, 1985, [29]	1980–1982	America	Argentina	70,768	38
Castilla-15, 1985, [29]	1980–1982	America	Bolivia	8,514	5
Castilla-16, 1985, [29]	1980–1982	America	Brazil	43,702	26
Castilla-17, 1985, [29]	1980–1982	America	Colombia	1,623	0
Castilla-18, 1985, [29]	1980–1982	America	Chile	25,634	23
Castilla-19, 1985, [29]	1980–1982	America	Ecuador	19,463	10
Castilla-20, 1985, [29]	1980–1982	America	Paraguay	3,443	2
Castilla-21, 1985, [29]	1980–1982	America	Peru	15,943	4
Castilla-22, 1985, [29]	1980–1982	America	Uruguay	10,916	11
Castilla-23, 1985, [29]	1980–1982	America	Venezuela	55,828	35
Groisman-2, 2017, [70]	2009–2013	America	Argentina	703,422	212
Forrester-1, 1998, [71]	1987–1996	America	USA	150,000	75
Parks, 2011, [72]	1999—2005	America	USA	2,594,295	677
Cragan-1, 2009, [73]	1995–2004	America	USA	470,802	81
Besser, 2007, [74]	1968–2003	America	USA	398,165	434
de Souza, 2020, [75]	2012–2017	America	Brazil	30,761	9
James, 1993, [30]	1970–1987	America	USA	15,487,449	6040
Parker-1, 2010, [76]	2004–2006	America	USA	3,120,605.00	697
Parker-2, 2010, [76]	2004–2006	America	USA	2,075,973	211
Parker 3, 2010, [76]	2004–2006	America	USA	2,145,287	192
Feuchtbaum, 1999, [77]	1990–1994	America	USA	1,618,279	770
Windham-1, 1982, [78]	1966–1972	America	USA	865,351	447
Aguiar, 2003, [79]	1999–2000	America	Latin-America	18,807	24
Poletta, 2018, [80]	1990–2013	America	Venezuela	353,956	155
Castilla-24, 2003, [81]	1999	America	Chile	10,740	10
Castilla-25, 2003, [81]	2000	America	Chile	12,977	5
Castilla-26, 2003, [81]	2001	America	Chile	11,462	7
Forrester-2, 2000, [82]	1986–1997	America	USA	246,189	89
Winsor, 1986, [83]	1980–1984	America	Canada	61,500	43
Bidondo, 2015, [84]	2009–2013	America	Argentina	703 325	164
De Wals, 2007, [85]	1993–2002	America	Canada	1,909,741	830
Yang, 2004, [86]	1989–2000	America	USA	2,615,197	617
Boulet-1, 2011, [87]	1995–2005	America	USA	522,315	29
McBride, 1979, [88]	1952–1970	America	Columbia	686,326	466
Siffel, 2005, [89]	1978–2001	America	USA	874,100	243
Mathews-1, 2002, [90]	1991	America	USA	3,564,453	655
Mathews-2, 2002, [90]	1992	America	USA	3,572,890	457
Mathews-3, 2002, [90]	1993	America	USA	3,562,723	481
Mathews-4, 2002, [90]	1994	America	USA	3,527,482	387
Mathews-5, 2002, [90]	1995	America	USA	3,484,539	408
Mathews-6, 2002, [90]	1996	America	USA	3,478,723	416
Mathews-7, 2002, [90]	1997	America	USA	3,469,667	434
Mathews-8, 2002, [90]	1998	America	USA	3,519,240	349
Mathews-9, 2002, [90]	1999	America	USA	3,533,565	382
Mathews-10, 2002, [90]	2000	America	USA	3,640,376	376
Mathews-11, 2002, [90]	2001	America	USA	3,649,061	343
Cragan-2, 1995, [91]	1985–1994	America	USA	211,024	268
Canfield, 2009, [92]	1999–2003	America	USA	1,827,317	514
Feldman, 1982, [93]	1968–1976	America	USA	173,655	89
Naggan-1, 1969, [94]	1930–1933	America	USA	14,052	38
Naggan-2, 1969, [94]	1934–1937	America	USA	16,179	28
Naggan-3, 1969, [94]	1938–1941	America	USA	18,206	34
Naggan-4, 1969, [94]	1942–1945	America	USA	22,059	25
Naggan-5, 1969, [94]	1946–1949	America	USA	28,097	25
Naggan-6, 1969, [94]	1950–1953	America	USA	43,441	37
Naggan-7, 1969, [94]	1954–1957	America	USA	52,032	32
Naggan-8, 1969, [94]	1958–1961	America	USA	57,639.00	35
Naggan-9, 1969, [94]	1962–1965	America	USA	60,002	51
Windham-2, 1982, [95]	1966–1979	America	USA & Norway	1,656,116	802
Boulet-2, 2008, [96]	1999–2000	America	USA	3,165,992	782
Boulet-3, 2008, [96]	2001–2002	America	USA	3,218,605	692
Boulet-4, 2008, [96]	2003–2004	America	USA	3,242,424	642
Bupp, 2015, [97]	1992–2012	America	USA	1,116,289	240
Nasri, 2014, [98]	1991–2011	Africa	Tunisia	3,803,889	174
Berihu, 2018, [99]	2018	Africa	Ethiopia	14,903	99
Forci, 2020, [100]	2011–2016	Africa	Morocco	43,923	22
Buccimazza, 1994, [101]	1973–1992	Africa	South Africa	516,252	164
Omer, 2016, [102]	2014–2015	Africa	Sudan	36,785	18
Riley, 1998, [103]	1983–1995	Australia	Australia	825,051	452
Owen, 2000, [104]	1983–1997	Australia	Australia	949,914	550
Chan-1, 1993, [105]	1966	Australia	Australia	20,556	24
Chan-2, 1993, [105]	1967	Australia	Australia	20,597	8
Chan-3, 1993, [105]	1968	Australia	Australia	21,424	27
Chan-4, 1993, [105]	1969	Australia	Australia	22,185	25
Chan-5, 1993, [105]	1970	Australia	Australia	22,817	13
Chan-6, 1993, [105]	1971	Australia	Australia	23,246	27
Chan-7, 1993, [105]	1972	Australia	Australia	22,073	25
Chan-8, 1993, [105]	1973	Australia	Australia	20,651	22
Chan-9, 1993, [105]	1974	Australia	Australia	20,417	22
Chan-10, 1993, [105]	1975	Australia	Australia	20,175	17
Chan-11, 1993, [105]	1976	Australia	Australia	19,157	15
Chan-12, 1993, [105]	1977	Australia	Australia	19,438	15
Chan-13, 1993, [105]	1978	Australia	Australia	18,736	17
Chan-14, 1993, [105]	1979	Australia	Australia	18,641	19
Chan-15, 1993, [105]	1980	Australia	Australia	18,638	20
Chan-16, 1993, [105]	1981	Australia	Australia	19,052	12
Chan-17, 1993, [105]	1982	Australia	Australia	19,128	19
Chan-18, 1993, [105]	1983	Australia	Australia	19,800	15
Chan-19, 1993, [105]	1984	Australia	Australia	20,281	17
Chan-20, 1993, [105]	1985	Australia	Australia	19,833	14
Chan-21, 1993, [105]	1986	Australia	Australia	19,800	16
Chan-22, 1993, [105]	1987	Australia	Australia	19,395	16
Chan-23, 1993, [105]	1988	Australia	Australia	19,530	14
Chan-24, 1993, [105]	1989	Australia	Australia	19,823	17
Chan-25, 1993, [105]	1990	Australia	Australia	19,988	23
Chan-26, 1993, [105]	1991	Australia	Australia	19,749	20
Barry Borman, 1986, [106]	1978	Australia	New Zealand	52,143	51
BORMAN, 1993, [107]	1978–1982	Australia	New Zealand	262,821	205

**Table 2:** Summary of study specifications (incidence of Anencephaly)

First author, year, references	Report year	Continent	Country	Sample size	Number of patients with Anencephaly
Safdar, 2007, [108]	1997–2005	Asia	Saudi Arabia	33,489	1
Al-Ani, 2010, [109]	2007–2008	Asia	Iraq	10,016	9
Bener, 2012, [110]	1985–2009	Asia	Qatar	302,049	102
Akar-1, 1988, [111]	1983	Europe	Turkey	628	1
Akar-2, 1988, [111]	1984	Europe	Turkey	563	1
Akar-3, 1988, [111]	1985	Europe	Turkey	756	2
Akar-4, 1988, [111]	1986	Europe	Turkey	1145	2
Akar-5, 1988, [111]	1987	Europe	Turkey	600	6
Onrat, 2009, [112]	2003–2004	Europe	Turkey	8631	12
SN ÍPEK, 2002, [113]	1961–1999	Europe	Czech Republic	5,499,008	1812
McDonnell-2, 2015, [114]	2009–2011	Europe	Republic of Ireland	226,923	106
Evans, 1979, [115]	1965–1976	Europe	Wales	70,871	146
Van Allen-1, 2006, [116]	1997	America	Columbia	44,734	17
Van Allen-2, 2006, [116]	1998	America	Columbia	43,141	12
Van Allen-3, 2006, [116]	1999	America	Columbia	42,040	28

**Table 3:** Summary of study specifications (mortality of Anencephaly)

First author, year, references	Report year	Continent	Country	Sample size	Number of deaths due to Anencephaly
Kancherla, 2018, [117]	2015	Asia	India	25,794,000	64,485
Tanner, 2010, [118]	1999–2006	America	USA	1,701,076	123
Wen-1, 2000, [119]	1981–1983	America	Canada	580,000	116
Wen-2, 2000, [119]	1993–1995	America	Canada	542,857	38
Dixon, 2019, [120]	2016	Africa	Ethiopia	3,328,867	21,638

The result of the I^2^ test for the prevalence of anencephaly in different parts of the world indicates a significant heterogeneity between studies (I^2^ = 99.9), so the data were analyzed by meta-analysis using a random effects model. Due to the high heterogeneity of the studies, sensitivity analysis was performed and the effect of each study on the final result and the degree of heterogeneity were evaluated. Based on Begg and Mazumdar rank correlation tests, the publication bias in the studies with less than 0.1% was not observed. (P = 0.105) (Table 4).

**Table 4:** General analysis of the prevalence of anencephaly per 10,000 births worldwide and continents by sample size, heterogeneity, publication bias

Meta-analysis	N	Sample size	I^2^	Begg and Mazumdar	Prevalence (95% CI)
Overall prevalence	340	169,407,738	99.9	0.105	5.1 (95% CI 4.7–5.5)
Continent					
Asia	50	12,449,402	99.9	0.776	6.5 (95% CI 5.5–7.7)
Europe	126	43,826,079	99.9	0.906	4.8 (95% CI 4.2–5.5)
America	128	106,111,868	99.9	0.809	4.3 (95% CI 3.8–4.8)
Africa	5	4,415,752	99.9	0.278	6.5 (95% CI 1–9.9)
Australia	30	12,615,064	99.7	0.111	8.6 (95% CI 7.7–9.5)

As a result of the combination of studies, the overall estimate of the prevalence of Anencephaly in the world will be 5.1 per ten thousand births (95% confidence interval 4.7–5.5) based on the random effects model (Table 4).

According to different reports of Anencephaly prevalence in different parts of the world, subgroup analysis by different continents (Asia, Europe, USA, Africa and Australia) is reported in Table 2, which has the highest prevalence in Australia with 8.6 per ten thousand births (confidence interval). 95%: 7.7–9.5) (Table 4).

Incidence and mortality of Anencephaly were 8.3 per ten thousand births (95% confidence interval 5.5–9.9) and 5.5 per ten thousand births (95% confidence interval 1.8–15) respectively (Table 5).

**Table 5:** General analysis of the incidence and mortality of anencephaly per 10,000 births worldwide and continents by sample size, heterogeneity, publication bias

Continent	N	Sample size	I^2^	Begg and Mazumdar	Prevalence (95% CI)
Incidence	15	6,284,594	99.9	0.766	8.3 (95% CI 5.5–9.9)
Mortality	5	31,946,800	99.9	0.462	5.5 (95% CI 1.8–15)

## Discussion

Neural tube defects (NTDs) are a major congenital structural disorder of the brain and spinal cord that occurs early in pregnancy as a result of defective neural tube closure [9], including abortion, stillbirth, and lifelong disability, as well as high emotional, psychological and economic consequences (138). Many factors, including radiation therapy, drugs, malnutrition, chemicals, and genetic determinants (mutations in folate-responsive or folate-dependent pathways) can adversely affect CNS growth during pregnancy and cause neural tube defects [12].

Anencephaly, which is the partial or complete absence of the brain and skull [3] is one of the most common forms of NTD. The fetus with anencephaly dies or will die in the first few hours after birth [9]. Exposure to methotrexate, aminopterin and valproic acid, maternal characteristics, race, ethnicity, geography, nutritional, biological and poor economic conditions are all risk factors for anencephaly [121, 122].

According to the present systematic review and meta-analysis, the overall prevalence of anencephaly in the world was 5.1 per ten thousand births. The highest prevalence of anencephaly was related to the study of RICHARDS et al. [57] with 230.69 infants with anencephaly per ten thousand births and the lowest prevalence was related to the study of Castilla et al. [31] with zero cases per ten thousand births. The most comprehensive study in terms of sample size was the study of James et al. (1993) with 15,487,449 people in the USA [32] that reported the prevalence of anencephaly at 3.89 per thousand births. Also, the present study estimated the risk of incidence and death due to anencephaly: 8.3 per ten thousand births and 5.5 per ten thousand births worldwide. Bhide et al. (2013) reported the prevalence of anencephaly in India at 2.1 per thousand births through 19 studies [123]. A meta-analysis and systematic review by Bitew et al. (2020) reported the prevalence of NTD in Ethiopia. 63.3 per ten thousand births [124]. Our study is almost in line with these studies and regarding the cause of minor differences between the present study and these studies, we can point out that the number of articles studied in the present study is more (121 articles in the present study versus 19 articles in the study of Bhide et al.) And also, the present study has examined patients with different races and geographical regions in the world.

Due to the change in population structure in different countries and different reports of the prevalence of anencephaly, the need for a detailed study of the prevalence of this defect in different continents in order to pay more attention to the process and its consequences seems inevitable. Therefore, according to the analysis of subgroups according to different continents, the highest prevalence of anencephaly is related to the continent of Australia with 8.6 per ten thousand births and the lowest belongs to the Americas with 4.3 per thousand births.

The results show that in addition to genetics, various environmental factors can also be involved in the development of anencephaly. So far, folic acid is the most important factor in preventing neural tube defects. Reports suggest the use of periovulation fulate supplements significantly reduces the risk of recurrence of anencephaly and other neural tube defects [125].

Regarding the serious nature of anencephaly and its high mortality, genetic counseling, folic acid supplements and prenatal diagnosis of neural tube defects are extremely important or (Given the seriousness of anencephaly and its high mortality rate, genetic counseling, folic acid supplements, and prenatal detection of neural tube abnormalities are critical.). This defect can be diagnosed by screening AFP (alpha-fetoprotein) with a combination of ultrasound and amniocentesis between 14 and 16 weeks of gestation [3, 5]. These studies can provide useful information to health care providers and enrich health care interventions and improve the quality of services and life [126].

## Limitations

One of the limitations of this study is that some samples were not based on random selection. Also, non-homogeneous reporting of articles, non-homogeneous method of implementation, and unavailability of the full text of the papers presented at the conference can be added. Such conditions can justify the high heterogeneity reported in the studies, and therefore, if these limitations and differences in the studies did not exist, the heterogeneity analysis could be less.

## Conclusion

The results of this study demonstrate that the prevalence of anencephaly in the world is high; therefore, it is necessary for physicians and specialists to emphasize the importance of preventive as well as control and treatment strategies.

## Data Availability

Datasets are available through the corresponding author upon reasonable request.

## Abbreviations

NTDs: Neural Tube Defects
SID: Scientific Information Database
MESH: Medical Subject Headings
WoS: Web of Science
PRISMA: Preferred Reporting Items for Systematic Reviews and Meta-Analysis
STROBE: Strengthening the reporting of observational studies in epidemiology for cross-sectional study
